# Carbon sink–source dynamics influence bud awakening under warming and defoliation

**DOI:** 10.1093/plphys/kiaf451

**Published:** 2025-09-30

**Authors:** Laura Fernández-de-Uña

**Affiliations:** Assistant Features Editor, Plant Physiology, American Society of Plant Biologists; Department of Plant Biology and Soil Sciences, Universidade de Vigo, Ourense 32004, Spain

Plant metabolism largely depends on carbohydrates. Nonstructural carbohydrates (NSCs; i.e. those not constituting biomass) are a key energy source and serve as the initial components of many structural and metabolic compounds as well as they regulate cell osmotic potential (Hartmann and Trumbore 2016). The most basic NSCs are the isomers glucose and fructose, which are synthesized during photosynthesis and are either directly consumed or transformed into different metabolites, including structural compounds, such as cellulose, and reserve compounds, such as sucrose or starch as well as lipids and proteins. Soluble sugars are transported across the plant through the phloem, mainly in the form of sucrose, to be used in cell metabolism or stored as starch. In addition to sugars, polyols (i.e. chemically reduced sugars or sugar alcohols), such as D-pinitol, play an important role in plant carbon transport and storage, as they are chemically inert and thus not as easily metabolized as sugars (Merchant and Richter 2011). The interconversion among the different NSCs largely depends on the plant's metabolic needs.

Plant tissue NSC concentrations depend on the balance between carbon supply (sources) and demand (sinks). One of the major carbon sinks is growth. Perennial plants, such as trees, adapt the renewal of their tissues and organs (e.g. leaves) to periods with favorable conditions, which define species phenology. Growth phenology is partly regulated by the activation of different phytohormones and the availability of resources such as water and carbon (Pantin et al. 2012). Warmer temperatures in the spring, for example, significantly advance growth reactivation (Delpierre et al. 2016). In addition to their structural and osmotic functions in new tissue formation, sugars, particularly sucrose, have been suggested to play a key role in signaling bud dormancy release (Wingler 2018). Besides abiotic and internal signals, biotic factors such as herbivore-induced defoliation may affect plant photosynthetic and carbon storage capacity, in turn affecting plant phenology and growth. However, how the interaction among these cues affects tree phenology remains largely unexplored.

In a recent *Plant Physiology* article, Deslauriers et al. (in press) explored how warming (+2 °C) and defoliation caused by eastern spruce budworm (*Choristoneura fumiferana*) affected the interplay among bud phenology, apical and lateral branch, internode and needle features, and NSC allocation in balsam fir (*Abies balsamea*) and black spruce (*Picea mariana*; Fig. 1).

**Figure 1.**
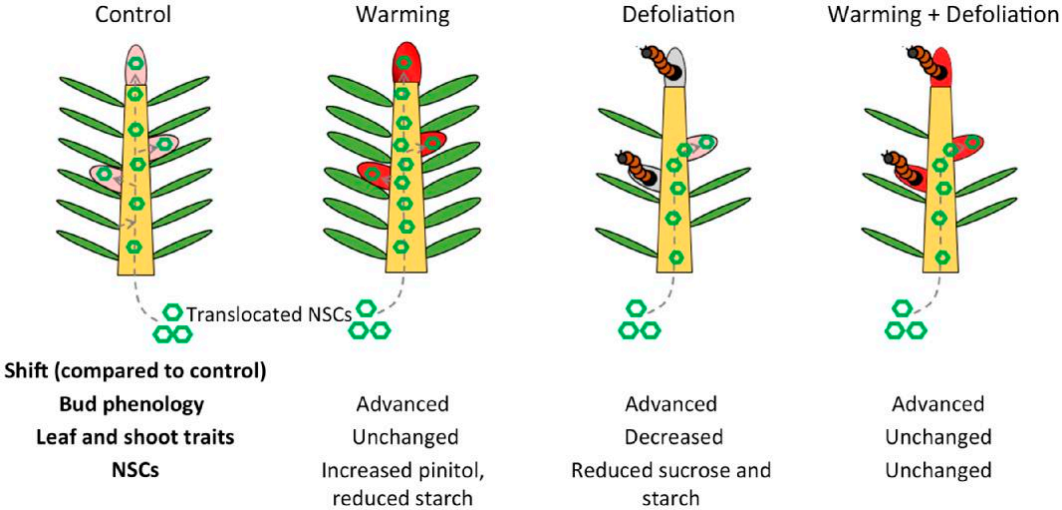
Main results obtained by Deslauriers et al. (in press). Leaf and shoot traits measured were leaf area, specific leaf area, number of needles, and shoot volume. Adapted from Deslauriers et al. (in press).

In black spruce, the phenology of lateral buds was significantly advanced by all treatments, with trees subjected to warming and defoliation opening buds the earliest, followed by warming alone and defoliation alone (Deslauriers et al. in press). Conversely, apical bud phenology was advanced only under warming. In balsam fir, the advance in bud phenology was significant only for the warming–defoliation treatment for lateral buds, while the phenology of apical buds was similarly advanced in all 3 treatments as compared with the control.

All treatments presented similar NSC dynamics along bud development, with the greater variations being found in storage compounds (pinitol, sucrose, and starch; Deslauriers et al. in press). Fructose decreased in all organs while starch increased, consistent with the conversion of soluble sugars into starch during spring bud dehardening, prior to growth reactivation. Sucrose did not change along bud phenologic phases in needles and twigs, while it decreased in buds, suggesting constant conversion to glucose and fructose to maintain metabolism and build new needles. Conversely, pinitol increased in buds but decreased in needles, indicating the translocation of this compound, via the phloem, from older needles to the forming buds (Dominguez and Niittylä 2022). The increase in pinitol concentrations may further indicate the use of this compound to regulate cell osmotic potential, inducing water absorption and thus increasing turgor and facilitating cell expansion (Merchant and Richter 2011; Pantin et al. 2012). Pinitol synthesis may also prevent photosynthesis downregulation induced by the accumulation of glucose and fructose in the forming buds (Merchant and Richter 2011).

Deslauriers and colleagues found no significant effect of warming in the different leaf and shoot traits assessed—namely, needle number and area, specific leaf area (i.e. the ratio of leaf area to leaf dry mass), and shoot internode wood volume. This suggests that the advancement in bud phenology due to warming was not translated into shifts in carbon allocation to bud and shoot growth (Deslauriers et al. in press). Therefore, the reduction in starch concentrations observed under warming may be associated with greater carbon consumption by respiration (Dusenge et al. 2019) rather than needle growth. Warming increased pinitol content, especially in buds (Deslauriers et al. in press). Increased pinitol concentrations may have thus offset the negative effect that temperature-induced water deficits could have caused on cell growth through osmoregulation (Turner 2018). However, whether the increase in pinitol is a protective response to warming or has an active role in regulating bud phenology needs to be further evaluated.

As a result of defoliation, earlier bud phenology was associated with a lower number of existing and current needles, lower specific leaf area, and smaller shoots. Defoliation decreased the number of buds requiring carbon (sinks); thus, the reduced competition for resources and need to replace damaged needles may have prompted an earlier bud dormancy break (Deslauriers et al. in press), albeit at the expense of producing smaller needles due to limited carbon availability. Indeed, defoliation caused a reduction in sucrose and starch concentrations as compared with control trees, suggesting greater consumption of available carbon for the reparation and replacement of damaged buds. The reduction in photosynthetic area may have further hindered defoliated tree capacity to synthesize and mobilize sucrose and store excess carbon as starch.

A greater frequency of insect outbreaks is expected under climate change scenarios because of the positive effect that warmer temperatures have on insect populations, particularly defoliators (Johnson and Haynes 2023). While warming can have positive effects on trees (e.g. advanced phenology), greater temperatures may increase heat and drought stress, rendering trees more vulnerable to pest attacks. The study by Deslauriers et al. sheds light on the effect of warming and defoliation on conifer bud phenology, contributing to our understanding and predictive capacity of the effects of climate warming on tree vulnerability to defoliator pests.

## Data Availability

No new data were generated or analysed in support of this article.
